# Differences between horse selection based on two forms of osteochondrosis in fetlock

**DOI:** 10.1007/s13353-018-0437-6

**Published:** 2018-03-09

**Authors:** D. Lewczuk, A. Bereznowski, M. Hecold, M. Frąszczak, A. Ruść, A. Korwin-Kossakowska, J. Szyda, S. Kamiński

**Affiliations:** 1 Institute of Genetics and Animal Breeding PAS, ul.Postępu 36A, 05-552 Magdalenka, Poland; 2 Warsaw University of Life Science-SGGW, ul.Nowoursynowska 166, 02-787 Warsaw, Poland; 3 University of Warmia and Mazury, ul.Oczapowskiego 5, 10-719 Olsztyn, Poland; 4 University of Environmental and Life Science, ul.CK Norwida 25/27, 50-375 Wrocław, Poland

**Keywords:** Horse, Osteochondrosis definition, GWAS, Selection

## Abstract

Horses lose potential opportunities because of health problems. Available breeding strategies are not effective enough, probably also because of the different definition used and its genetic usefulness. The aim of the study was to compare the genetic background estimated by the genome-wide association study (GWAS) for osteochondrosis using two different scaling osteochondrosis (OC)/healthy and osteochondrosis dissecans (OCD)/healthy systems for evaluating the disease status of investigated fetlock joints. Two hundred one Warmblood horses trained for performance tests (87 stallions and 114 mares) were phenotyped and genotyped. Four fetlock x-ray images per horse were collected using the RTG Girth HF 80 and Vet Scan ray 3600. The DNA of each horse was genotyped using the BeadChip 70K. To identify SNPs that significantly affect the probability of osteochondrosis, two different methods were applied: the Cochran-Armitage test based on an additive mode of inheritance and logistic regression. The genetic background for osteochondrosis, expressed in the number of SNPs found with significant associations with osteochondrosis, was higher by evaluation in the scale of OCD/healthy horses (16 SNPs on several chromosomes mainly on the ECA1 and ECA10) than OC/healthy (2 SNPs on the ECA15 and one SNP on the ECA10). Detailed definition of osteochondrosis is needed in breeding and in veterinary practice. The genetic background for osteochondrosis and osteochondrosis dissecans seems not the same. Suggestive SNPs could be the candidate markers for osteochondrosis but should be checked on a larger population before usage.

## Introduction

For a long time, osteochondrosis selection has been carried out by sport horse associations. The results do not seem satisfactory enough as even after 30 years of selection the occurrence of affected horses has not decreased (Koenen et al. 2000; Stock and Distl 2006; Hilla and Distl 2014). A lot of investigations were carried out on osteochondrosis selection reviewed by van Grevenhof (2011), Distl (2013) and Lewczuk and Korwin-Kossakowska (2012). Heritability in equine reached the values from 0.2 to 0.64 and depends on breed and location. Genotyping achieved by using Illumina Equine SNP50 BeadChip with 54,602 SNPs showed high call rate from 98 to 99.8%. Obtained results demonstrate from 11 to 172 highly polymorphic microsatellites on different chromosomes (Bates et al. 2014). There are many breeding strategies available, but first of all a clear, proper and unique definition of osteochondrosis is needed. Most theories are based on the thesis that a failure of the cartilage canal’s blood supply caused by ischemic necrosis of chondrocytes leads to osteochondrosis latens and its future possible development into osteochondrosis manifesta and osteochondrosis dissecans. First, osteochondrosis was introduced as developmental orthopaedic disease (McIlwraith 2004). One of the simplest definitions of osteochondrosis involves the term osteochondrosis dissecans (OCD) when a loose bone fragment is present and osteochondrosis (OC) for lesser defects where such fragments have not yet been formed (Van Weeren 2006). The perception of investigated forms of osteochondrosis is not always presented in detail (Lepeule et al. 2013). Nowadays, presentation of osteochondrosis can be simplified as a failure of endochondral ossification (McCoy et al. 2016). Proper definition of disease seems of special importance as even some cases of osteochondrosis may become clinically undetectable also until horses are put into training (Van Weeren 2006). On the other hand, when trauma or physical stress is involved in the primary induction of osteochondrosis dissecans-like lesions, the diagnosis is controversial (McIlwraith 2004). Depending on the orthopaedic treatment, location and size, different scales can be used (McIlwraith 2013).

In recent years, discussions have been conducted on the heritability of osteochondrosis. It has been underlined that a wider scale produces higher heritability and the possibility of successive horse selection (van Grevenhof 2011); however, some authors have pointed out that there is a low correlation between flattening and osteochondral fragments, so flattening might be simply a misinterpretation of normal counter varieties (Lykkjen et al. 2010, 2013). For horse breeding practitioners, the effectiveness and transparency of the selection criteria are essential. It seems necessary in the overall discussion on osteochondrosis selection to search for the genetic background of the condition specified by different definitions. The lack of agreement in previous gene mapping studies may reflect confounding due to variability in phenotypic criteria for OC (McCoy et al. 2016). The hypothesis of the current research is that the genetic background will be the same for osteochondrosis and osteochondrosis dissecans. The aim of the study was to compare the results of the genome-wide association study (GWAS) for osteochondrosis using two different scaling systems—OC/healthy and OCD/healthy horses—for evaluating the status of the disease for investigated fetlock joints.

## Material and methods

### Horses and training

Two hundred one Warmblood horses tested during two successive years (87 stallions and 114 mares) were phenotyped and genotyped. The sample population comprised all individuals tested at official performance tests conducted in two training centres in Poland. Investigated horses were at the mean age of 1305 days (1047–1722) and bred by private and studs breeders. They were pre-selected on the basis of their conformation, with an average height of 165 cm at the withers (156–174); a chest circumference of 190 cm (156–207); and a cannon bone circumference of 20.75 cm (18.50–22.50). The mean conformation score of the evaluated horses was 78 points (75–84) on a scale of 0–100. The horses originated from 129 sires. Three of the sires had more than 5 offspring, 15 sires had 3 and 4 offspring and 23 sires had 2 offspring. The other 88 sires had only a single offspring in the investigated group of horses. All horses belonged to opened and closely related sport breeds (studbooks registers). Fourteen sires had progeny in two different breeds. Earlier analysis of the overall horse osteochondrosis dissecans health status showed the heritability 0.27–0.30 (Lewczuk et al. 2017). Both performance tests for young horses were based on basic training and conditioning at official training stations, conducted by the Polish Horse Breeders Association according to standardised conditions. Horses were trained in riding and jumping during performance tests, while stallions were additionally trained in stamina skills. They worked 6 days per week. The daily workload did not exceed 45 min. The performance tests for young horses took 100 days for stallions and 60 days for mares.

### Roentgen images and scaling systems

Horses were x-rayed at the end of the tests. Four images per horse were collected using an RTG Girth HF 80 and Vet Scan ray 3600. The fetlock joints were analysed (metacarpophalangeal and metatarsophalangeal). The following locations were evaluated in the fetlock joint: the sagittal ridge of the metacarpal/metatarsal condyle, the dorsal margin of the proximal phalanx and the plantar margin of the proximal phalanx. Images were evaluated as blind by a two-person vet team according to a real selection breeding system so that horses were selected, on a binary scale, as positive or negative for osteochondrosis in two different scales. The first scaling divides horses as being without/with signs of osteochondrosis (manifesta stages evaluated as positive). The following disorders were taken into account as osteochondrosis (OC): focal loss and condensation of the bone shadow in the place of the closure of the epiphyseal growth plate, bone formation in the place of attachment of the synovial capsule, flattening of the bone shadow in the pre-cartilage zone, clear loss of the bone shadow in the pre-cartilage zone and clear loss of the bone shadow in the pre-cartilage zone characterised by a cavity in the bone’s proper borderline. The second system divides horses as having or not having loose bone fragments and therefore, only osteochondrosis dissecans (OCD), the last stage of disease, was taken into account. Such investigations of the data allow a comparison to be made of the genetic background of different clinical stages of osteochondrosis for individual joints.

The osteochondrosis data of the studied group of horses were taken from the report of an earlier project (NR 12 0037 06 for the Polish Horse Breeders Association). In calculations for fetlock joints, the following rates of horses were healthy: 4% in the OC scaling and 76% in the OCD scaling.

### Blood samples and SNP genotyping

Blood samples were collected according to standard veterinary procedures. For DNA extraction, blood samples from the 201 animals were collected into 10-ml tubes containing potassium ethylenediaminetetraacetic acid as an anticoagulant and stored at − 78 °C. Genomic DNA was isolated from blood samples with the MasterPure Genomic Purification Kit (Epicentre, USA), and its quantity and quality were evaluated by NanoDrop measurements and standard agarose electrophoresis. Next, the DNA of each horse was genotyped using 70K Illumina Neogen Equine Array (GeneSeek). The quality of SNP clusters was analysed using GenomeStudio (version 2011.1, Illumina, San Diego, CA) software. The average call rate was 99.75%.

### Statistical analysis

A data analysis of associations between SNPs and evaluations of osteochondrosis was performed according to different evaluation systems (OC/healthy and OCD/healthy). From the set of 65,157 SNPs after quality control, 56,811 SNPs were selected for further analysis. The SNP selection criteria that were applied comprised polymorphism with a minimum minor allele frequency (MAF) of 0.01 and a minimum call rate of 90% within the analysed sample of horses. To identify SNPs significantly affecting the probability of osteochondrosis in horses, two different methods were applied. Statistical analysis included the Cochran-Armitage test and logistic regression, assuming an additive model of inheritance in the Cochran-Armitage test defined as

$$ T=\left({\mathrm{NS}}_{\mathrm{AB}}\mathrm{NH}-{\mathrm{NH}}_{\mathrm{AB}}\mathrm{NS}\right)+2\left({\mathrm{NS}}_{\mathrm{BB}}\mathrm{NH}-{\mathrm{NH}}_{\mathrm{BB}}\mathrm{NS}\right) $$

where NS represents the number of horses diagnosed as OC/OCD positive, NH represents the number of horses which were OC/OCD free and the subscripts AB and BB correspond to a heterozygous and one of the homozygous SNP genotypes, respectively. Asymptotically under H0, the statistic transformed as $\frac{T}{\sqrt{\mathrm{Var}(T)}}$ follows the standard normal distribution, with the variance defined as

$$ \mathrm{Var}(T)=\frac{\mathrm{NS}-\mathrm{NH}}{N}\cdot c, $$

where

$$ c=\left({\mathrm{NS}}_{\mathrm{AB}}+{\mathrm{NH}}_{\mathrm{AB}}\right)\left(\mathrm{N}\hbox{-} {\mathrm{NS}}_{\mathrm{AB}}-{\mathrm{NH}}_{\mathrm{AB}}\right)+4\left({\mathrm{NS}}_{\mathrm{BB}}+{\mathrm{NH}}_{\mathrm{BB}}\right)\left(\mathrm{N}\hbox{-} {\mathrm{NS}}_{\mathrm{BB}}-{\mathrm{NH}}_{\mathrm{BB}}\right)-\left({\mathrm{NS}}_{\mathrm{AB}}+{\mathrm{NH}}_{\mathrm{AB}}\right)\left({\mathrm{NS}}_{\mathrm{BB}}+{\mathrm{NH}}_{\mathrm{BB}}\right) $$

The applied logistic regression model had the following form:

$$ \mathrm{logit}\left({P}_{\mathrm{sick}}\right)={\beta}_1{X}_{\mathrm{SNP}}+{\beta}_2{X}_{\mathrm{training}\_\mathrm{centre}}+{\beta}_3{X}_{\mathrm{sex}}+{\beta}_4{X}_{\mathrm{breed}}+{\beta}_5{X}_{\mathrm{age}}+{\beta}_6{X}_{\mathrm{breed}\mathrm{er}}+{\beta}_7{X}_{\mathrm{sire}}+{\beta}_8{X}_{\mathrm{dam}} $$

where *β*_*i*_, *i* = 1, 2, …, 8 are the regression coefficients, and the explanatory variables denote the genotype of SNP (coded as 0 for homozygous, 1 for heterozygous and 2 for alternative homozygous SNP), training centre (two levels of this variable), sex (two levels), breed register (6 levels), age (in days), kind of breeder (two levels), sire and dam. The probability that an animal is sick is denoted by *P*_sick_ and the logit function is the natural logarithm of odds: $1\mathrm{n}\left(\frac{P_{\mathrm{sick}}}{1-{P}_{\mathrm{sick}}}\right)$. Functions of the R package were used.

Because of the relatively low heritability of the trait, SNPs (and corresponding genes) of very high effects on the occurrence of OCD are not expected. Therefore, we decided to present in the table suggestive SNPs which reach nominal significance, but not significant after multiple testing correction. Note that the Bonferroni correction is too conservative for several reasons including the fact that it assumes the independence of each test even though many of the SNPs are in linkage disequilibrium and thus correlated with each other.

## Results

The number of SNPs that were statistically significant for the orthopaedic evaluation was higher for osteochondrosis treated in the scaling system as OCD/healthy than for the scoring of any symptoms of OC/full healthy horses. The hypothesis about the same genetic background of OC and OCD in the real selection is not confirmed. All SNPs that were statistically significant for both scaling systems for each investigated joint are presented in Tables 1 and 2.

**Table 1:** SNPs statistically significant for the evaluation of OCD for the fetlock joint (metacarpal phalangeal/metatarsal phalangeal)

System OCD (only osteochondrosis dissecans treated as ill)		
SNP identification	*P* value	Position (ECA) Mb
BIEC2_22135	4.20E−02	(1) 52.99
BIEC2_490019	3.17E−02	(2) 76.23
BIEC2_1008454	7.8E−04	(7) 82.05
BIEC2_102646	3.17E−02	(10) 7.89
BIEC2_130298	2.05E−07	(10) 66.96
BIEC2_130301	2.05E−07	(10) 66.96
BIEC2_141465	2.34E−02	(11) 3.24
BIEC2_408453	2.03E−03	(18) 20.52
BIEC2_409553	3.49E−02	(18) 26.44
BIEC2_430250	2.80E−02	(19) 15.53
BIEC2_524840	9.91E−04	(20) 23.37
BIEC2_630333	2.34E−02	(24) 6.21
BIEC2_1116623	2.37E−02	(X) 26.85
BIEC2_29595	1.46E−02	(1) 70.95
BIEC2_30646	1.51E−02	(1) 69.45
BIEC2_579755	1.05E−02	(22) 8.49

BIEC2: names of the SNPs given by the Illumina BeadChip producer

ECA equine chromosome

**Table 2:** SNPs statistically significant for the evaluation of OC for the fetlock joint (metacarpal phalangeal/metatarsal phalangeal)

System OC (all signs of illness treated as ill)		
SNP identification	*P* value	Position (ECA) Mb
BIEC2_137996	2.25E−02	(10) 72.68
BIEC2_323837	4.10E−02	(15) 82.17
BIEC2_323907	1.96E−02	(15) 82.29

Several SNPs were found statistically significant for OCD evaluation for the fetlock. SNPs found statistically significant for osteochondrosis dissecans were located on ECA1, ECA2, ECA7, ECA10, ECA11, ECA18, ECA19, ECA20, ECA22, ECA24 and ECAX. Only three SNPs were found significant for the osteochondrosis (OC) evaluation. Two of them were located on ECA15 and one on ECA10. Some of the analysed SNPs were located within the functional genes, but they were mostly introns of these genes. It means they are not directly related to the transfer of genetic information.

## Discussion

A comparison of our results with other results obtained earlier can be made on the basis of the genomic position of a given SNP since two different types of BeadChip were used (70K and 50K). No exactly identical SNPs could be found for the fetlock in comparison with the results of Corbin et al. (2012). SNPs on the same chromosomes associated with osteochondrosis in the fetlock were reported by Lykkjen et al. (2013). The data cited above are compared with our results (Table 3).

**Table 3:** Comparison of SNP positions statistically significant for OCD in various studies and our results

ECA	SNPs at positions [Mb]	Author	Our SNPs at positions [Mb]	Joint
1	160.5	Lykkjen et al. 2013	70.95; 69.45	Fetlock
	139.7	Lykkjen et al. 2013		Fetlock
3	105.2–105.8	Orr et al. 2012		Hock
	113.5	Teyssedre et al. 2012		Hock
	103.4–107.9	Teyssèdre et al. 2012		Hock
	88.5	Corbin et al. 2012		Stifle
4	39.26	Corbin et al. 2012		Hock
5	42.5 77.4 79.3	Lykkjen et al. 2010		Hock
7	69.6 80.5	Lykkjen et al. 2013	44.3	Fetlock
15	87.3	Teyssedre et al. 2010	82.17–82.29	Fetlock
10	60.5 80.3–80.4	Lykkjen et al. 2010		Hock
	48.25–48.33	Orr et al. 2012		Hock
11	10.9	Lykkjen et al. 2013	3.24	Fetlock
14	67.97–77.90	Teyssèdre et al. 2012		Hock
18	36.77	Corbin et al. 2012		Hock

More data are available in the literature for the evaluation of the hock joint. SNPs that were significant according to Teyssèdre et al. (2010, 2012) were located on chromosomes ECA3 and ECA14. According to Corbin et al. (2012), the only SNP significant for most of the evaluated joints was located on ECA3 at 88.5 Mb and the results of Orr et al. (2012) indicated more significant SNPs associated with osteochondrosis on this chromosome at 105.2–105.8 Mb. The other chromosome with important SNPs identified in cited studies is the ECA10. The study by Lykkjen et al. (2010) suggested chromosomes as containing significant SNPs on ECA5, ECA10, ECA27, ECA28. The widest genetic background, the highest number of SNPs associated with osteochondrosis found for the hock joint, was observed in the study by Teyssèdre et al. (2010). This study indicated a similar number of SNPs affecting osteochondrosis in the fetlock (12 SNPs) as in our study.

The data for the SNP analysis of the stifle joint was presented by Corbin et al. (2012). The SNP that was found to be significant by these authors was at 88.49 Mb on ECA3. Some of the SNPs cited in the above literature support our result (Teyssèdre et al. 2010, 2012) for the fetlock joint. For example, on ECA15, position 87.3 Mb (at the significant region 86.3–89.75 Mb) was found to be associated with “global measurement of osteochondrosis” and “fetlock measurement for osteochondrosis”. Our results showed that positions 82.17 and 82.29 Mb on the same chromosome were statistically significant for the fetlock. In the cited paper on the basis of a haplotype analysis, also chromosome ECA13 was significant for osteochondrosis in general and in the fetlock in two regions 0.22–12.88 Mb.

It seems necessary to distinguish in detail what kind of definition for osteochondrosis is investigated in each paper. It is noteworthy that each of the above-mentioned studies used a different clinical definition of osteochondrosis. In the cited works, Teyssèdre et al. (2012) recorded every symptom of osteochondrosis as a lesion on the basis of size and consistency. Lykkjen et al. (2013) classified POF (phalanx/plantar osteochondral fragment) on the basis of the position in the joint (lateral/medial), and no further details about POF recognition were given. Corbin et al. (2012), on the other hand, investigated OCD positive/negative classification, while Orr et al. (2012) used the conventional Dutch classification, which scores RTG images on the A–E scale. “A” indicates normal bone contour and “D” and “E” indicate fragments. Lykkjen et al. (2010) evaluated their horses for OC and OCD; however, in the statistical analysis, the scale of OCD-affected/unaffected horses was used. The data of Teyssèdre et al. (2010) recorded a global and local horse score on the basis of osteochondrosis type anomaly and severity. In the case of the OCD affected/unaffected classification used by Lykkjen et al. (2010, 2013), the most significant number of SNPs was found to be the same also for the OC/healthy classification. However, this was not the case in the study by Corbin et al. (2012) using the same scaling system (OCD/healthy). According to our results, the genetic background seems to be higher for scaling the osteochondrosis as OCD like OC. Such results may be caused by the fact that the OCD scaling is more obvious and easier to be correctly recognised than the OC scaling. Correct and precise interpretation may be the main key to osteochondrosis selection. The other possibility for osteochondrosis selection can be provided by quite new definitions of the disease and other groupings of the diseases, as has been introduced lately (Denoix et al. 2013; Ricard et al. 2013); however, the possibility of incorrect disease interpretation increases with the number of the diseases included into the new description. Obtained results give information on the kind of selection that might be more useful in the horse breeding. Based on our results, the selection according to the scaling “osteochondrosis dissecans/healthy” gives wider genetic background than the selection “osteochondrosis/healthy”. Even if the amount of horses was not high in our study, it was exactly the same in both groups, with the same genetic structure for the OC and OCD groups. Suggestive SNPs could be used as the candidate markers for osteochondrosis after verification on a larger population. The selection based on OCD evaluation gives the promise for a rapid genetic progress. Such results might be connected with overestimation of the OC symptoms mentioned earlier; however, it should be confirmed on a larger population. Furthermore, a large part of horses being defined as healthy in regard to OCD were affected by OC; thus, the control groups did not consist of horses completely free from any sign of disease in both analyses. However, that is the way they are selected by breeders. The other treatment of experimental groups was even not possible because of the low numbers of horses completely free from osteochondrosis.

The genetic background for osteochondrosis, expressed in the number of SNPs associated with the disease that are found to be significant, was higher in the evaluation of the disease in the scale based on the occurrence of osteochondrosis dissecans (OCD/healthy horses) than of osteochondrosis (OC/healthy). It seems necessary to keep a detailed description of the scale of osteochondrosis evaluation to allow a comparison of the results as the definition of the osteochondrosis influenced the results. The description of the disease should be as clear as possible especially from the breeders’ point of view, as they should be able to understand the selection they make as much as possible. The clarification of the criteria in accordance with genetic background is needed for the effective selection of sport horse populations.
